# Multimodal Lung Cancer Subtyping Using Deep Learning Neural Networks on Whole Slide Tissue Images and MALDI MSI

**DOI:** 10.3390/cancers14246181

**Published:** 2022-12-14

**Authors:** Charlotte Janßen, Tobias Boskamp, Jean Le’Clerc Arrastia, Daniel Otero Baguer, Lena Hauberg-Lotte, Mark Kriegsmann, Katharina Kriegsmann, Georg Steinbuß, Rita Casadonte, Jörg Kriegsmann, Peter Maaß

**Affiliations:** 1 Center for Industrial Mathematics (ZeTeM), University of Bremen, 28359 Bremen, Germany; 2 Bruker Daltonics, 28359 Bremen, Germany; 3 Institute of Pathology, University Hospital Heidelberg, 69120 Heidelberg, Germany; 4 Translational Lung Research Center Heidelberg (TLRC), Member of the German Center for Lung Research (DZL), 69120 Heidelberg, Germany; 5 Institute of Pathology Wiesbaden, 65199 Wiesbaden, Germany; 6 Department of Hematology, Oncology and Rheumatology, University Hospital Heidelberg, 69120 Heidelberg, Germany; 7 Proteopath, 54926 Trier, Germany; 8 Center for Histology, Cytology and Molecular Diagnostic, 54296 Trier, Germany; 9 Faculty of Medicine and Dentistry, Danube Private University, 3500 Krems, Austria

**Keywords:** deep learning, artificial intelligence, lung cancer, mass spectrometry imaging, non-small cell lung cancer, whole slide images, tumor detection, tumor segmentation

## Abstract

**Simple Summary:**

For the effective treatment of lung cancer patients, correct tumor subtyping is of utmost importance, but it is often challenging in clinical routine. Using artificial intelligence and combining information from digital microscopy and matrix-assisted laser desorption/ionization mass spectrometry imaging data have the potential to support the pathologist’s decision-making process. We present a classification algorithm to distinguish between adenocarcinoma and squamous cell carcinoma of the lung based on the automatic detection of tumor areas in whole tissue sections and the determination of the tumor subtype with high accuracy.

**Abstract:**

Artificial intelligence (AI) has shown potential for facilitating the detection and classification of tumors. In patients with non-small cell lung cancer, distinguishing between the most common subtypes, adenocarcinoma (ADC) and squamous cell carcinoma (SqCC), is crucial for the development of an effective treatment plan. This task, however, may still present challenges in clinical routine. We propose a two-modality, AI-based classification algorithm to detect and subtype tumor areas, which combines information from matrix-assisted laser desorption/ionization (MALDI) mass spectrometry imaging (MSI) data and digital microscopy whole slide images (WSIs) of lung tissue sections. The method consists of first detecting areas with high tumor cell content by performing a segmentation of the hematoxylin and eosin-stained (H&E-stained) WSIs, and subsequently classifying the tumor areas based on the corresponding MALDI MSI data. We trained the algorithm on six tissue microarrays (TMAs) with tumor samples from N = 232 patients and used 14 additional whole sections for validation and model selection. Classification accuracy was evaluated on a test dataset with another 16 whole sections. The algorithm accurately detected and classified tumor areas, yielding a test accuracy of 94.7% on spectrum level, and correctly classified 15 of 16 test sections. When an additional quality control criterion was introduced, a 100% test accuracy was achieved on sections that passed the quality control (14 of 16). The presented method provides a step further towards the inclusion of AI and MALDI MSI data into clinical routine and has the potential to reduce the pathologist’s work load. A careful analysis of the results revealed specific challenges to be considered when training neural networks on data from lung cancer tissue.

## 1. Introduction

The correct classification of the most common non-small cell lung cancer (NSCLC) types, adenocarcinoma (ADC) and squamous cell carcinoma (SqCC), is essential for mutational testing and subsequent treatment decisions. An accurate diagnosis, however, often involves evaluating several immunohistochemical (IHC) tissue stainings, which requires a substantial amount of the pathologist’s time. Moreover, due to limited availability of tissue, such multiple stainings are not always feasible.

The application of artificial intelligence (AI) in digital pathology has shown great potential for various diagnostic tasks [1,2,3], including lung cancer tumor subtyping [4,5,6]. Likewise, matrix-assisted laser desorption and ionization (MALDI) mass spectrometry imaging (MSI) has been successfully used for tumor typing in the past decades [7,8,9,10,11], in particular for the subtyping of neoplastic lung tissue [12,13,14]. Additionally, the combination of MALDI MSI data and machine learning techniques has made great advances in recent years [15,16,17,18,19]. The first AI-based algorithm to classify peptide imaging data was presented by Behrmann et al. [20].

Machine learning techniques typically require large data cohorts that show a high biological variation and hence these methods are usually developed and evaluated on tissue micro arrays (TMAs), the construction of which is not feasible in standard clinical routine. Recently, we presented an AI-based classification algorithm for discriminating ADC and SqCC of the lung based on MALDI MSI data from whole tissue sections [21]. This method, however, still requires a pathologist to annotate tumor areas. The automatic detection of tumor areas in whole slide images (WSIs) was recently presented for skin cancer [22] with the use of an adaptation of a U-Net neural network [1].

In this study, we present a bimodal tumor typing method combining information from WSIs of lung tissue sections and corresponding MALDI MSI data. The network architecture from Le’Clerc Arrastia et al. [22] was used for the segmentation of the WSIs to detect areas with high tumor cell content. Subsequently, the respective co-registered MALDI MSI data were classified into ADC and SqCC using a previously developed neural network [20,21].

The fully automated algorithm detected and classified tumor regions in ADC and SqCC samples with high accuracy. Our results show that the tumor segmentation is suitable not only for a subsequent classification, but also to support the generation of training data. A careful visual analysis of the segmentation results revealed specific challenges in the classification of neoplastic lung tissue with AI-based techniques.

## 2. Materials and Methods

### 2.1. Tissue Samples

**Figure A1:**
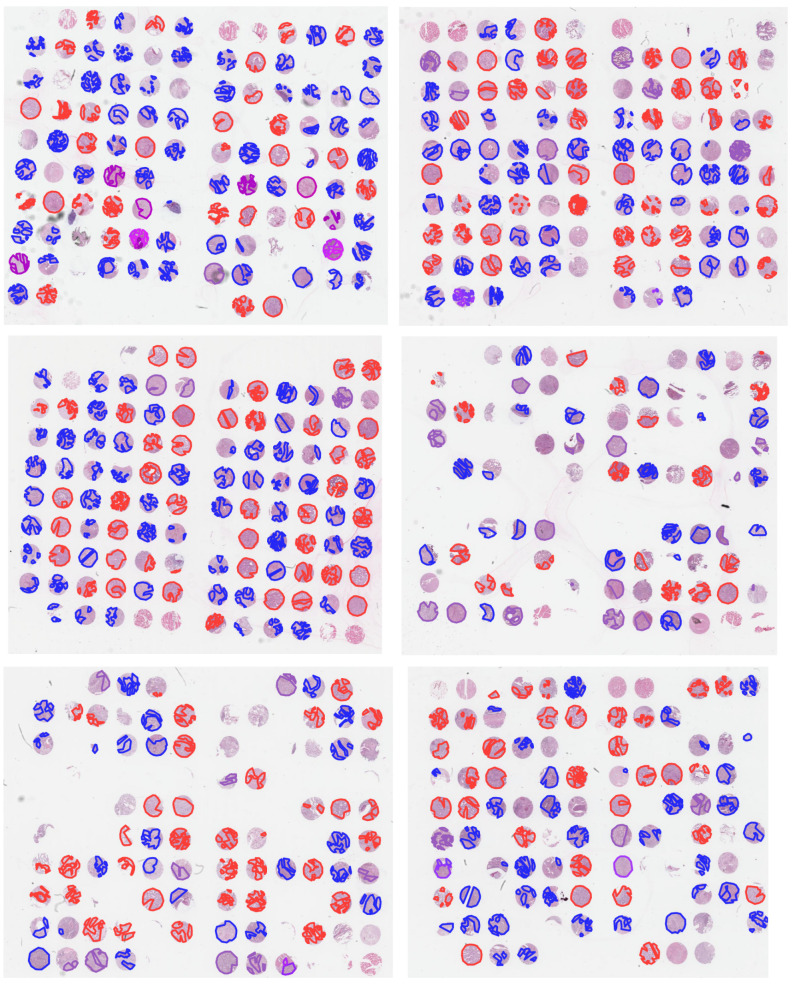
Hematoxylin and eosin (H&E)-stained images of TMAs with overlaid annotations (red lines: ADC, blue lines: SqCC, purple lines: other tumor types). Image was previously published in the work of Janßen et al. [21].

**Figure A2:**
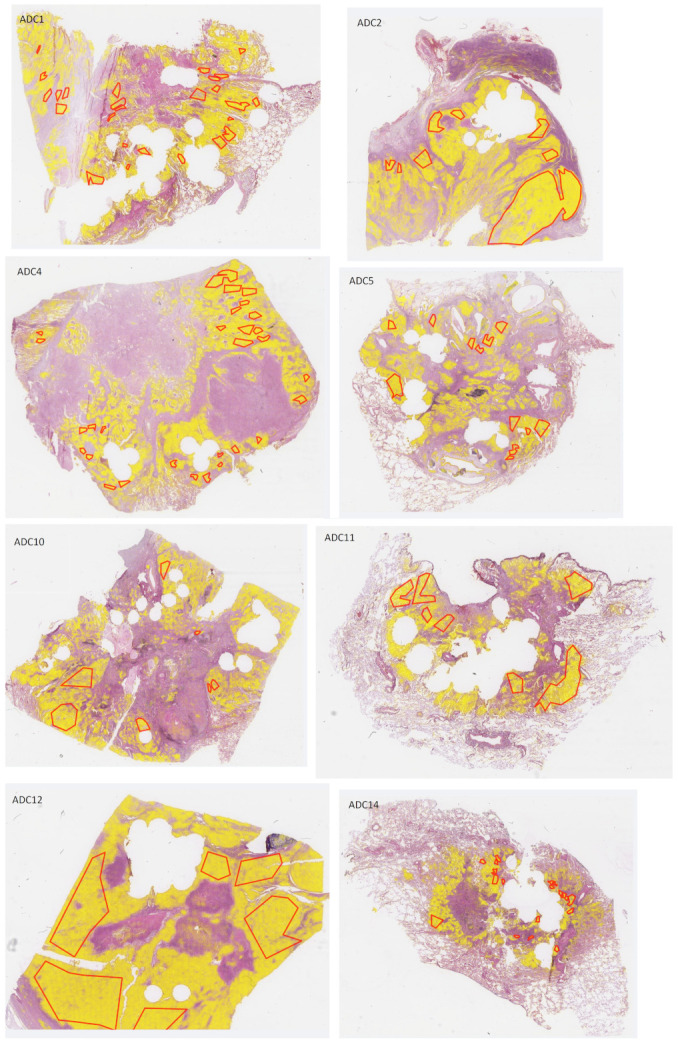

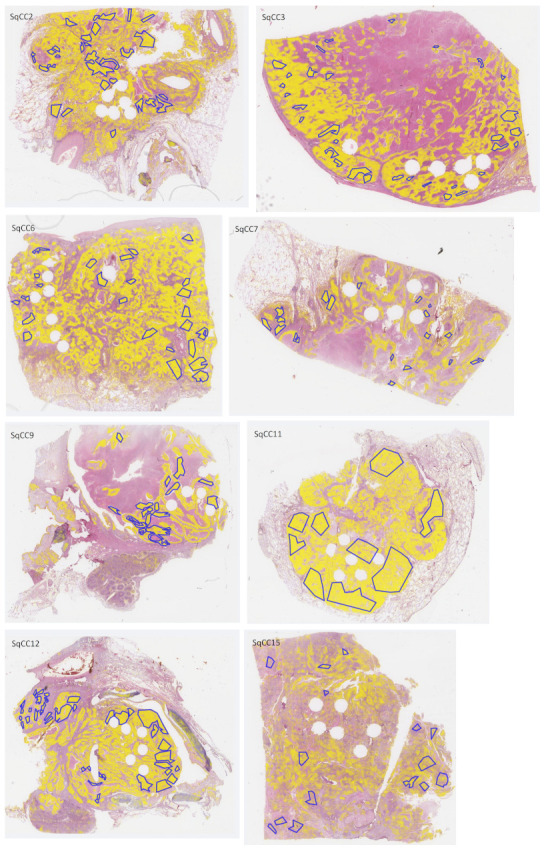
H&E-stained whole-slide images of test sections with overlaid annotations (red lines: ADC, blue lines: SqCC) and heatmaps (yellow filling) of segmented tumor areas based on network **Seg1**.

The study cohort comprised of 6 TMAs of NSCLC tissue cores and whole sections of ADC (N =15) and SqCC (N =15). The TMAs were assembled using the Tissue Biobank from the National Center of Tumor Diseases. The assembly of the TMAs was randomized, and all TMAs contained similar numbers of cores from both tumor entities (Figure A1 and Figure A2 in Appendix A).

The MALDI MSI measurements were described in detail in [19], and an overview is given below. Following the removal of the matrix, the TMAs and whole sections were hematoxylin and eosin (H&E)-stained and scanned by a slide scanner (Aperio AT2) at 40× magnification. Diagnoses for each core and each whole section were provided based on IHC stainings of serial sections (CK5/6, TTF1, Napsin and p40). Cores diagnosed with neither ADC nor SqCC and cores with ambiguous IHC staining results (CK5/6- and p40-negative SqCC cores and TTF1-negative ADC cores) were excluded, resulting in N = 179 cores of ADC and N = 223 cores of SqCC from a total of 232 patients (max. 2 cores per patient). The 30 whole sections were taken from 30 additional patients.

Areas with high tumor cell content, high scan quality, and low amount of necrosis were annotated by a thoracic pathologist. As the annotation of all tumor areas in a whole section would have been too time-consuming, only incomplete annotations on the whole sections were carried out, including only exemplary tumor areas (Figure A2).

#### MALDI MSI Measurements

The tissue sections were mounted onto indium tin oxide-coated glass slides. After dewaxing and rehydration, a heat-induced antigen retrieval was performed. For digestion, a trypsin solution (0.025 μg/μL final concentration) was applied with an automatic reagent sprayer (TMsprayer, HTX Technologies). After matrix application (10 mg/mL alpha-cyano-4-hydroxycinnamic acid) with the same spraying device, MALDI MSI was performed using a rapifleX MALDI Tissuetyper (Bruker Daltonics) in positive reflector mode. The measurements were carried out using flexImaging (version 5.0, Bruker Daltonics) and flexControl(version 4.0, Bruker Daltonics). More details on the MALDI MSI measurements can be found in [19].

MALDI MSI data were imported into the SCiLS Lab software (version 2018b, Bruker Daltonics), and convolutional baseline correction was applied. Subsequently, the SCiLS Lab API (version 1.0.554, Bruker Daltonics) was used to import the data into self-written Python code for all further processing.

**Figure A3:**
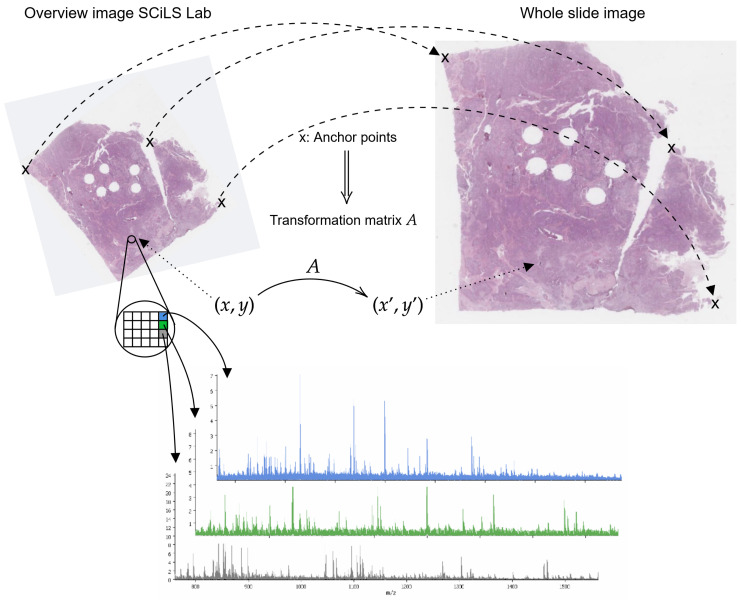
Sketch of image registration process. Anchor points (x) are used to compute transformation matrix *A*, which maps SCiLS Lab coordinates (x,y) to coordinates of whole slide images $\left(x^{\prime },y^{\prime }\right)$.

The MALDI data and the optical images needed to be co-registered into the same coordinate system. For each slide, we compared the overview image saved by SCiLS Lab with the WSI visualized in Python and then set anchor points on the same spots in both images (Figure A3). We used the scikit-image Python package [23] to compute an affine transformation that would map the MSI coordinates onto the WSI coordinate system.

### 2.2. Classification Algorithm

**Figure 1:**
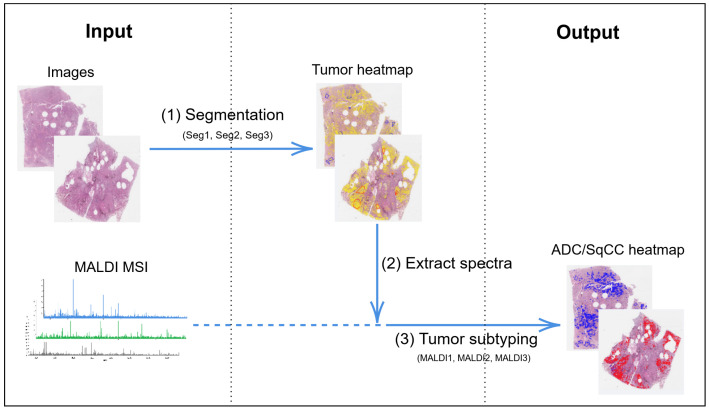
Overview of classification algorithm. Seg1, Seg2, Seg3 and MALDI1, MALDI2, MALDI3 refer to the different trained networks, cf. Section 2.2.2. MALDI MSI: matrix-assisted laser desorption/ionization mass spectrometry imaging, ADC: adenocarcinoma, SqCC: squamous cell carcinoma.

The classification algorithm consists of two parts (Figure 1). First, to detect areas with high tumor cell content, a segmentation of the WSIs was performed (1), i.e., a pixel-wise classification into classes tumor and non-tumor based on a neural network with a U-Net architecture [1,22]. Next, spectra from within the tumor regions were extracted (2), preprocessed [24], and classified into the two tumor subtypes (3) by a second neural network called IsotopeNet [20,21].

#### 2.2.1. Neural Networks and Preprocessing

A neural network is a mathematical function that receives a data point (in this case, an image patch or a spectrum) and outputs a class prediction. Neural networks learn by seeing training data for which the correct class is known. During training, the network’s output is compared to the actual class for each data point. A loss function measuring the quality of the network’s classification is computed, and the network’s internal parameters are adapted such that the loss function is continuously reduced. The networks are trained for several epochs, and the network sees the complete training data in each epoch.

The U-Net is a fully convolutional network for image segmentation and has achieved great results on medical image datasets [1]. In this study, we used an adapted version of the U-Net architecture [22] developed for the segmentation of tumor areas in the WSIs of skin specimen. The IsotopeNet was specifically designed for peptide imaging data [20]. Here, we used the slightly adapted architecture from our previous study [21]. More information about the architectures, training details, and hyperparameters for both networks can be found in the Appendix (Table A1).

Prior to the classification by the IsotopeNet, the spectral data were preprocessed to reduce technical variability [24,25], which, in our previous study, largely improved the classification performance [21]. The preprocessing pipeline includes the reduction of the m/z range to 700–2700 Da, an intensity profile normalization [25], a statistical recalibration to reduce mass shift [26], a peptide mass resampling [25], a spatial smoothing, a second intensity profile normalization, and an intensity log transformation.

#### 2.2.2. Experimental Design

In addition to the training dataset, validation and test datasets are required for the training and evaluation of a neural network. The validation dataset is used to tune hyperparameters and to choose the best network from all epochs. Since the training process is influenced by random effects in initialization and batch sampling, each training was repeated five times. Among all epochs and repetitions, the networks with the best performance on the respective validation dataset were finally chosen. What ‘best performance’ means depends on the specific metric used for evaluation, which in turn depends on the data modality (cf. Section 3). An independent test dataset is required to evaluate the performance of the final network. The choice of data for training, validation, and testing depends on the respective networks for segmentation or tumor subtyping.

##### Tumor Segmentation

The segmentation requires digital images as input and annotations of tumorous areas as ground truth for training, validation, and testing. Due to the incompleteness of the whole-section annotations, a quantitative evaluation of the whole sections is not possible. For this reason, we used a three-fold cross-validation to train and validate the segmentation networks exclusively on the TMAs. The six TMAs were randomly assigned to one of three groups, with two TMAs each. In each cross-validation, a network was trained on two groups and validated on the third group, which yielded three segmentation networks (Seg1, Seg2, Seg3, cf. Table 1). The 30 whole sections served as the test dataset, which only allowed a qualitative comparison.

Since there were far more pixels from one class (normal tissue) than from the other (tumor), we used the intersection over union (IoU) to evaluate the segmentation, which is more meaningful for very unbalanced datasets than, for example, the accuracy. The segmentation results were visualized as heatmaps, which indicated the probability of a pixel being part of a tumor region, and were carefully visually analyzed by a thoracic pathologist (M.K.).

##### Tumor Subtyping

The MALDI networks for tumor subtyping require spectra from tumorous regions as input and a diagnosis (ADC or SqCC) as ground truth for training, validation, and testing. We closely followed the process of our previous study [21], where we used the identical datasets and network architecture. There, we found that it is important to include whole sections in the validation dataset for the classification to be successful [21]. Therefore, we trained the MALDI networks on spectra from the 6 TMAs, validated them on 14 whole sections (7 of ADC and 7 of SqCC, randomly selected), and used the remaining 16 whole sections (8 of ADC and 8 of SqCC) for testing.

To investigate the suitability of using the segmentation as the basis for extracting relevant spectra, we compared three different MALDI networks that had been trained and validated on different subsets of relevant spectra (Table 1). The first (MALDI1) was the network from our previous study [21]. We used the pathologist’s annotations to extract relevant spectra for both training and validation. The second network (MALDI2) was trained on spectra from within the original annotations but validated on tumor spectra as detected by the segmentation (Seg1). Even though the training dataset was the same as that used for MALDI1, due to random effects, the training was not identical, and additionally, the validation dataset influenced the choice of the final network. The third network (MALDI3) was trained and validated on spectra from within tumor regions as defined by the segmentation. For each group of two TMAs, the training spectra were extracted from the tumor areas as defined by the segmentation network that had been validated on these TMAs. For example, as network Seg1 was validated on TMAs 2 and 6, spectra from TMAs 2 and 6 were extracted from the tumor regions as defined by network Seg1. The validation of MALDI3 was again carried out on spectra of the whole sections from areas as defined by Seg1.

To further examine the quality of the automatic segmentation and its suitability to identify relevant tumor areas, the three MALDI networks were evaluated on four different test datasets. All of the four test datasets were based on the 16 test sections but contained spectra from different areas. The first, Test Dataset 1, contained spectra from within the original annotations. Test Dataset 2 contained spectra from within the tumor areas as defined by the segmentation (Seg1), and Test Dataset 3 contained spectra from the segmented areas without the originally annotated areas. Finally, Test Dataset 4 included spectra from non-tumor regions as defined by the segmentation. All training, validation, and test datasets and their respective numbers of spectra can be found in Table 2.

##### Quality Control

The IsotopeNet yields a per-spectrum classification, which is typically not desired in a clinical setting. We computed a core-wise or section-wise classification based on a majority vote and combined with quality control to indicate potentially inconclusive results. More specifically, each core or section was assigned the tumor type of the majority (more than 50%) of its spectra. Following Kriegsmann et al. [6] and Janßen et al. [21], we considered a classification inconclusive if the majority was less than 90%. In a clinical setting, such sections may be highlighted for a more careful analysis by the pathologist. The quality control threshold of *p* = 0.9 can be adjusted according to the requirements of the application at hand.

## 3. Results and Discussion

### 3.1. Tumor Segmentation

**Figure 2:**
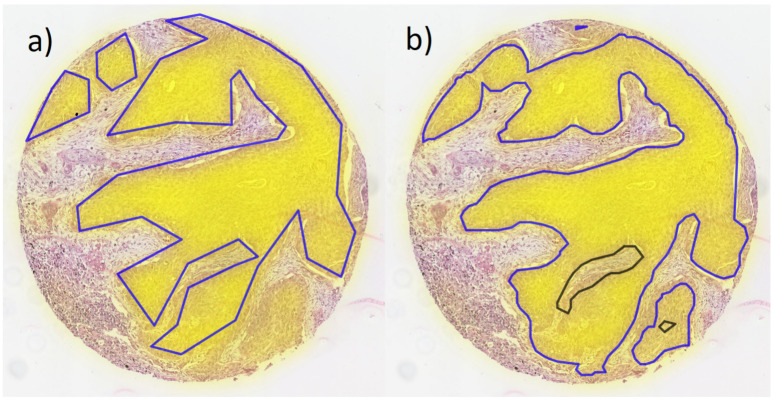
Example core from validation TMA with overlaid heatmap of segmentation (yellow filling); (**a**) original annotations of tumor areas and (**b**) tumor areas defined by segmentation (blue lines: tumor, black lines: normal tissue surrounded by tumor).

The segmentation network accurately detected most tumor areas on the TMAs (training and validation dataset) and even provided a refinement of the original annotations in many cases (Figure 2 and Table 3). Note that the quantitative evaluation of training and validation (Table 3) needs to be interpreted with caution, as the original annotations are not exact on pixel level. Thus, the IoU numbers may only partially reflect the true accuracy.

**Figure 3:**
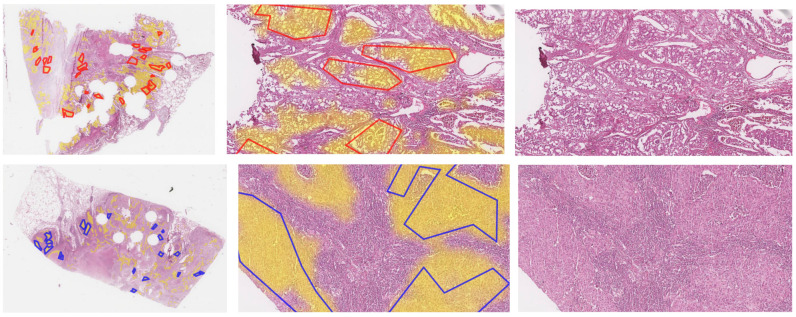
Whole slide images of test sections ADC1 and SqCC7, and zoomed-in images overlaid with original annotations of tumor areas (red lines: ADC, blue lines: SqCC) and heatmap of segmentation (Seg1, yellow filling). Images of all sections can be found in the Appendix A in Figure A2.

The segmentation detected tumor areas in the whole sections with high accuracy (Figure 3 and Figure A2 in Appendix A). Some regions with normal tissue were misclassified as a tumor area, and very few true tumor areas were segmented as normal. We identified several reasons for these misclassifications: Normal regions that were falsely segmented as a tumor area almost always belonged to cases with special cell structures, such as bronchial structures, peribronchial glands, macrophages, or areas with inflammation. In all of these special structures, certain similarities to neoplastic cells found in tumor areas can be observed, which may explain the tendency to misclassify such structures.

**Figure 4:**
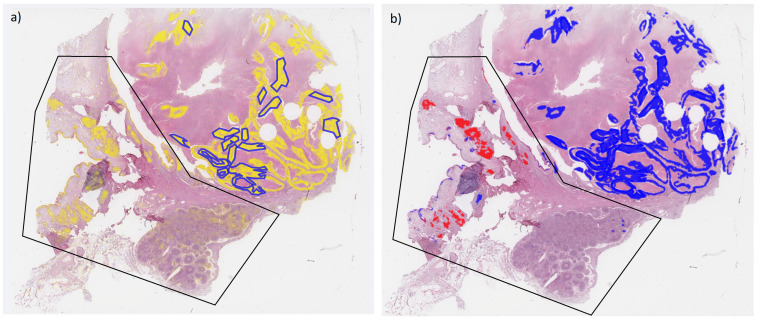
Whole slide image of test section SqCC9 overlaid with original annotations of tumor areas (blue lines). (**a**) Heatmap of segmentation (Seg1, yellow filling) and (**b**) heatmap of tumor classification (blue filling: SqCC, red filling: ADC). Black polygon highlights area with normal tissue misclassified as tumor.

In test section SqCC9, a relatively large area with normal tissue was misclassified as a tumor area (Figure 4a), including areas with a normal bronchial structure, respiratory epithelium, peribronchial glands (Figure A5I), and lymph nodes. The number of lymphocytes may be high in neoplastic tissue, especially at the invasion front, which is most likely the reason why the network segmented part of the lymphatic tissue as a tumor area. Furthermore, small areas of cartilage were falsely segmented as a tumor area; for example, in test section ADC5 (Figure A5C). In the H&E staining, these regions appeared to be darker than other normal regions, thus showing more similarity to tumor regions, which might be the reason for the misclassification. Tumor areas that were not detected are areas with low image quality, including coagulation artifacts, or areas with unusual staining properties.

In general, a neural network can only learn what is present in the training dataset, and it can only be as good as the ground truth annotations at least for a medium or small-sized dataset. To eliminate the previously mentioned misclassifications, the training dataset needs to contain many examples of special but normal tissue types, such as bronchial tissue, inflamed tissue, tissue with glandular structures, etc., and a range of atypical staining characteristics and artifacts. TMAs, on the other hand, are usually assembled from areas with high tumor cell content and good tissue preservation, which counteracts this objective.

### 3.2. Tumor Subtyping

**Figure A4:**
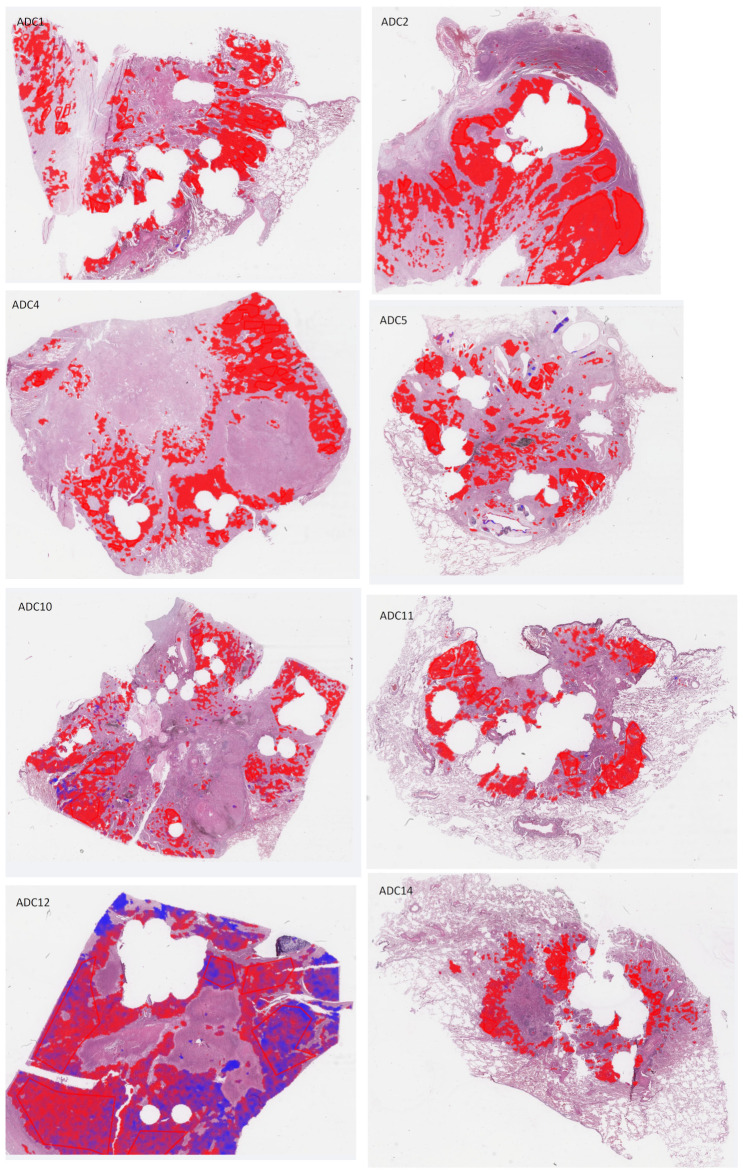

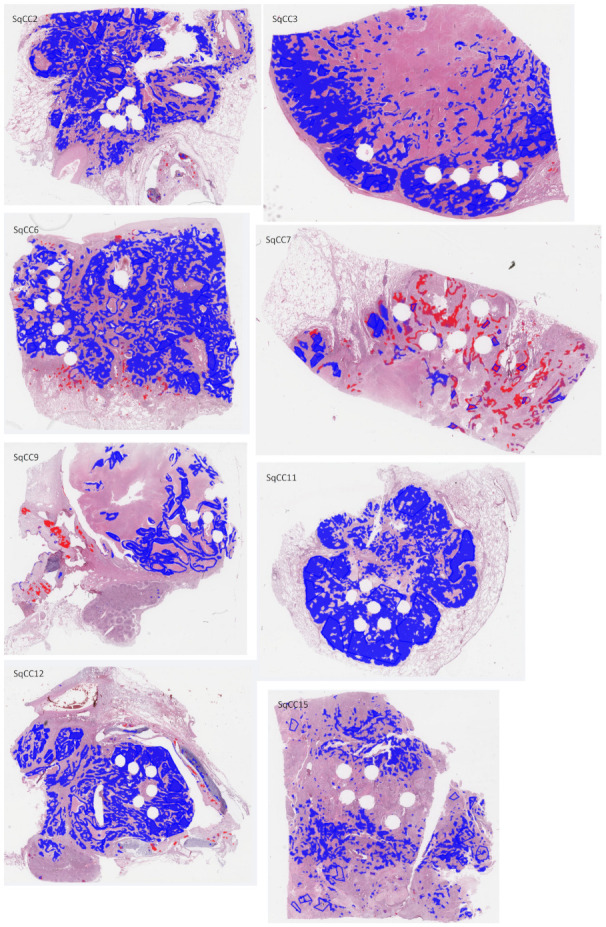
H&E-stained whole-slide images of test sections with overlaid annotations (red lines: ADC, blue lines: SqCC) and heatmaps (red filling: ADC, blue filling: SqCC) of the ADC/SqCC classification of network **MALDI2**.

All three MALDI networks showed high accuracy on the training and validation datasets (cf. Table A2 and Table A3 in the Appendix for spectra and core/section level results, respectively). Similarly, they achieved very good overall results on Test Datasets 1–3, both on spectra and section level (Table 4 and Table 5, cf. Table A4 in Appendix B for results without quality control, and Figure A4 in Appendix B).

A slightly better performance was achieved by network MALDI1 as compared to MALDI2 and MALDI3 on the spectra and section level for Test Datasets 1, 2, and 3. MALDI1 was trained and validated on the original annotations, which included only the most prominent tumor areas. Hence, this network saw the “cleanest” training dataset, which might have allowed it to learn the relevant features more easily. Another possible reason for MALDI1 being the best network overall might be that the hyperparameter tuning was performed for network MALDI1, which potentially represents a disadvantage for MALDI2 and MALDI3. However, since all three networks used the same architecture (and so theoretically, the optimal hyperparameters should be similar), we decided against individually tuning the hyperparameters for each network due to time limitations.

MALDI2 achieved slightly better results on Test Dataset 1 as compared to MALDI3 and very similar results on the other datasets. This is likely because Test Dataset 1, which contained only spectra from the original annotations, is most similar to the training dataset of MALDI2 (and MALDI1).

Additionally, it was observed that the results on Test Dataset 1 were slightly better compared to the results on Test Datasets 2 and 3 for networks MALDI1 and MALDI2. No significant difference was obtained on datasets 1–3 for the network MALDI3. First, the original annotations (Test Dataset 1) included only areas with high tumor cell content and were the most prominent tumor areas in each section. Hence, they were theoretically the easiest to classify (by humans or artificial intelligence). Second, normal tissue areas that were falsely segmented as tumor areas could have led to a false ADC/SqCC classification for Test Datasets 2 and 3 (example given below).

Substantially worse results were achieved on Test Dataset 4, which contained spectra from outside of the segmented areas. The accuracy of around 75% on Test Dataset 4 might be surprising, as one could have assumed that the classification of non-tumor spectra would be random and therefore yield accuracies of around 50%. The reason might be that the normal tissue still contained proteins (e.g., Cytokeratin 7) that could also be found in tumors, which then led to the “correct” classification of spectra from these areas regarding the tumor type of the respective section. Additionally, Dataset 4 contained spectra from some small tumor areas that were not detected by the segmentation.

### 3.3. Quality Control

**Figure A5:**
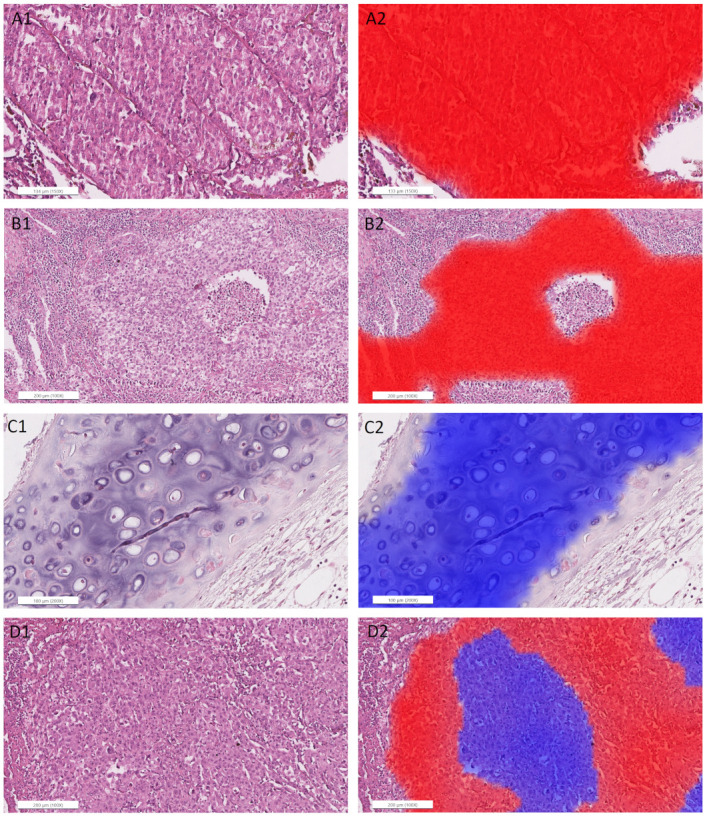

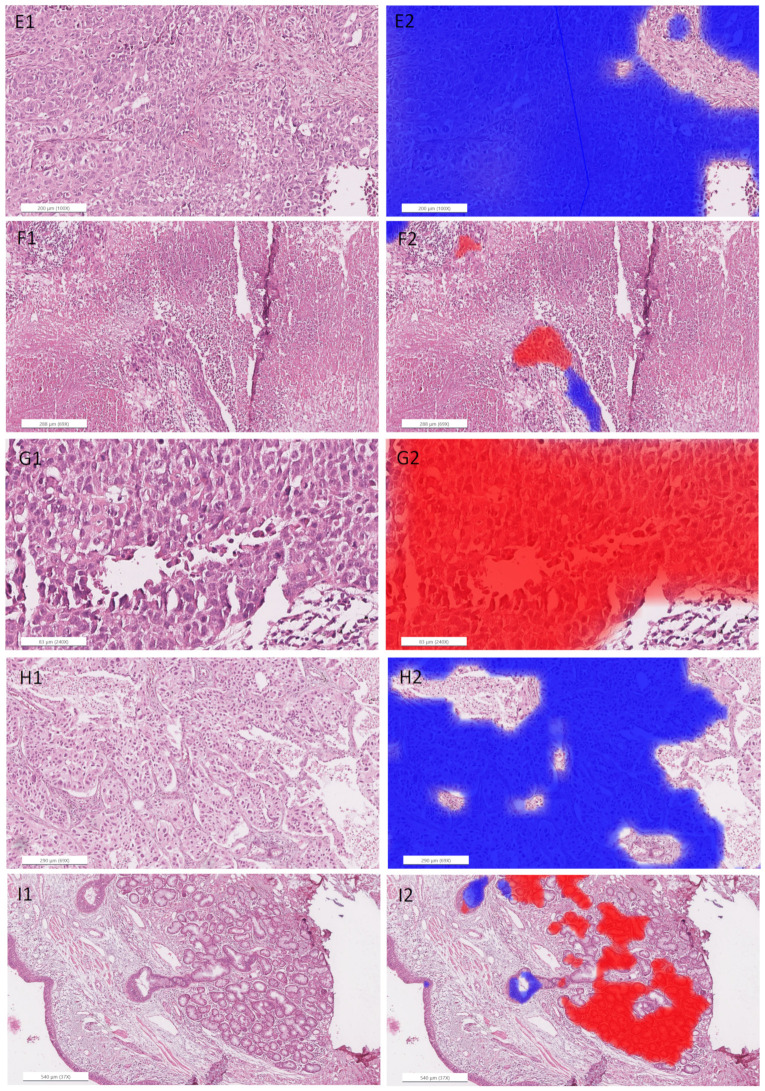
Detailed images of representative areas in test sections (**left**) overlaid with heatmaps (**right**; red filling: ADC, blue filling: SqCC) for the ADC/SqCC classification of network **MALDI2**. (**A**): ADC1, solid growth pattern; (**B**): ADC4, solid growth pattern; (**C**): ADC5, cartilage; (**D**): ADC12, pure solid growth pattern (middle) and subtle glandular structures (surrounding); (**E**): SqCC6, solid growth pattern; (**F**): SqCC7, necrosis and artifacts (cracks and tissue folding); (**G**): SqCC7, pseudo glandular structures and artifacts; (**H**): SqCC11, solid growth pattern; (**I**): SqCC9, peribronchial glands (**right**) and respiratory epithelium (**left**).

One particular section, SqCC7, did not pass the quality control for all networks and test datasets (Figure A4 and Figure A5F,G). The same result had already been observed in our previous study [21]. On closer investigation, we identified several problems in this section. Some areas with normal tissue were falsely segmented as tumor areas, most likely due to the inflammation of the tissue in these regions. These areas were, in turn, falsely classified as ADC. The actual tumor tissue in this section mainly showed a solid growth pattern with subtle glandular structures, partially caused by tissue artifacts. The section additionally contained large areas of necrosis. It is likely that spectra from necrotic areas did not contain tumor-specific peptide signals, which could have been the cause of the misclassification. Finally, this section exhibited some artifacts in the form of tissue folding and cracks, which might have also impacted the quality of the corresponding MALDI data.

For MALDI2 and MALDI3, an additional ADC section did not pass the quality control (section ADC12, Figure A4 and Figure A5D). This section showed a pure solid growth pattern of ADC in some areas, which potentially caused the false classification by the network. In other areas, subtle glandular structures were visible, which had been classified correctly by the network. Solid growth patterns can make the distinction between ADC and SqCC based on the image harder to obtain, but it is not clear how these affected the spectra. Overall, this section would be difficult to classify in a routine clinical setting and would in fact require IHC staining for a definite diagnosis. The test dataset included several cases with solid growth patterns of ADC and of SqCC, which would have been difficult to classify in a routine clinical setting. However, the algorithm was able to successfully predict the correct tumor type (Figure A5A,B,E,H).

In Test Dataset 3 (spectra from areas that were segmented as tumor areas but not included in the original annotations), one more SqCC section (SqCC9, Figure 4b) and Figure A5I failed the quality control for all networks. The same section passed the quality control for all networks for Test Dataset 2 (all segmented spectra) only by a small margin. In fact, the actual tumor areas in this section were classified correctly as SqCC. Some normal tissue areas, however, were incorrectly segmented as tumor areas and subsequently misclassified as ADC.

### 3.4. Using Tumor Segmentations for Training

Overall, the results for all automatically detected tumor regions (Test Dataset 2) are very promising. Only minor differences were found when comparing these results to Test Datasets 1 or 3, which implies that spectra extracted from the segmentation are generally suitable for tumor classification.

Moreover, there are no substantial differences in the results achieved by MALDI2 (trained on original annotations) and MALDI3 (trained on the segmented spectra). This suggests that the automatic segmentation also allows for the creation of annotations for new training data, for which only a diagnosis but no manual, detailed annotations are available. Of course, these automatically generated annotations need to be used with caution, and a pathologist’s annotations would always be the gold standard. On the other hand, manually annotating training data is often the limiting factor, and expanding the training dataset without increasing the cost for manual annotations may be a valuable option in improving the performance and robustness of the classification model.

## 4. Conclusions

We presented a fully automatic algorithm to accurately detect and classify tumor areas in whole sections of pulmonary ADC and SqCC. Automatic segmentation is suitable for the extraction of tumor areas to be used in a subsequent tumor classification based on MALDI MSI data, and it is appropriate for the generation of tumor annotations for new training data, for which no manual annotations are available. The tumor subtyping accuracy could likely be further improved by training on a more extensive dataset, including more normal tissue categories, for example, bronchial epithelium, peribronchial glands as well as tumors from more patients to account for the great biological variation. Additionally, novelty detection techniques, which are a way of filtering out data that the network has not seen in the training data, would possibly be beneficial extensions to both classification steps.

We would like to highlight two more aspects of this study. First, the segmentation was successful on tissue that was analyzed by MALDI MSI and that was only H&E-stained and scanned afterwards. Hence, only one section of tissue is necessary for the complete classification process, thereby saving tissue for further analyses. Second, the segmentation can provide additional guidance in selecting regions for subsequent macro- or micro-dissection and DNA extraction, or in estimating the percentage of the tumor area, which may be more objective and reproducible than a visually estimated value. Thus, we believe that the presented methods may contribute in different ways to support and speed up the pathologist’s work in a clinical setting.

## Figures and Tables

**Table 1:** Overview of networks trained for tumor segmentation and subtyping (ADC/SqCC), and their respective training and validation datasets. TMAs: tissue micro arrays.

Network	Training Based on	Validation Based on
**Segmentation of tumor areas**		
Seg1, Seg2, Seg3	4 TMAs, original annotations	2 TMAs, original annotations
**Tumor subtyping (ADC/SqCC)**		
MALDI1 [21]	6 TMAs, original annotations	14 whole sections, original annotations
MALDI2	6 TMAs, original annotations	14 whole sections, segmentation Seg1
MALDI3	6 TMAs, segmentation Seg1, Seg2, and Seg3	14 whole sections, segmentation Seg1

**Table 2:** MALDI MSI test datasets and their respective numbers of spectra. All four test datasets were based on the 16 test sections.

		Number of Spectra		
	Dataset Contains Spectra from Within	Total	SqCC	ADC
Test Dataset 1	Original annotations	105.553	53.136	52.417
Test Dataset 2	Segmented areas (by network Seg1)	459.776	260.577	199.199
Test Dataset 3	Segmented areas without original annotations	366.746	214.221	152.525
Test Dataset 4	Non-tumor areas (complement of Test Dataset 2)	1409.629	760.265	649.364

**Table 3:** **Intersection over union** (IoU) for the training and validation of the segmentation networks. Mean IoU and standard deviation of five identical training processes are displayed in brackets. Respective network with the highest validation IoU is chosen.

Network	Training	Validation
Seg1	0.818 (0.834 ± 0.038)	0.743 (0.732 ± 0.011)
Seg2	0.816 (0.869 ± 0.032)	0.663 (0.651 ± 0.010)
Seg3	0.824 (0.849 ± 0.030)	0.735 (0.723 ± 0.013)

**Table 4:** Balanced accuracies of different MALDI networks for different **test datasets** on **spectra level**. Note that the values are based on the assumption that all spectra included in each dataset represent the true tumor type of the respective section, neglecting the fact that Datasets 2, 3, and particularly 4 contain spectra from non-tumor regions.

	MALDI1 (Trained and Validated on Original Annotations)	MALDI2 (Trained on Original, Validated on Segmented Areas)	MALDI3 (Trained and Validated on Segmented Areas)
Test Dataset 1	0.990	0.973	0.941
Test Dataset 2	0.971	0.947	0.947
Test Dataset 3	0.967	0.941	0.949
Test Dataset 4	0.767	0.769	0.735

**Table 5:** Confusion matrices of section-wise classification of different MALDI networks for different **test datasets** on **section level** (Pred. = Prediction, quality control with ***p* = 0.9**).

		MALDI1			MALDI2			MALDI3		
	True/Pred.	SqCC	ADC	*p* < 0.9	SqCC	ADC	*p* < 0.9	SqCC	ADC	*p* < 0.9
Test Dataset 1	SqCC	7	0	1	7	0	1	7	0	1
	ADC	0	8	0	0	7	1	0	7	1
Test Dataset 2	SqCC	7	0	1	7	0	1	7	0	1
	ADC	0	8	0	0	7	1	0	7	1
Test Dataset 3	SqCC	6	0	2	6	0	2	6	0	2
	ADC	0	8	0	0	7	1	0	7	1
Test Dataset 4	SqCC	2	0	6	1	0	7	2	0	6
	ADC	0	2	6	0	7	1	0	2	6

## Data Availability

The data presented in this study are available on request from the corresponding author. The data are not publicly available due to privacy reasons.

## Abbreviations

The following abbreviations are used in this manuscript:

ADC	Adenocarcinoma
AI	Artifical intelligence
H& E	Hematoxylin and eosin
IHC	Immunohistochemical
IoU	Intersection over union
MALDI MSI	Matrix-assisted laser desorption/ionization mass spectrometry imaging
NSCLC	Non-small cell lung cancer
SqCC	Squamous cell carcinoma
TMA	Tissue microarray
WSI	Whole slide image

## Appendix A. Material and Methods

### Appendix A.2. Neural Networks

The U-Net [1] employed in this study is a fully convolutional neural network with a contracting and an expanding path as well as skip connections between the encoder and decoder blocks. Here, we used the ResNet34-Encoder, and the network was trained using deep supervision and linear merging with 5 blocks [22] as well as a focal loss [27]. Image patches of 1024 × 1024 pixels were extracted at 20× magnification. The input data were augmented through various image transformation techniques, such as rotation and blurring. The settings and choice of hyperparameters for the U-Net were based on empirical values.

The architecture of the IsotopeNet was specifically developed for peptide imaging data. It consists of two residual blocks including two convolutional layers each, a locally connected layer, and a fully connected layer [20]. A tuning of the hyperparameters was carried out in our previous study Janßen et al. [21].

All code was implemented in Python using the Pytorch library [28]. Experiments were performed on two different machines using either a 20-core processor (Intel Xeon Silver 4210) or a 16-core processor (AMD EPYC 7262), and 4 NVIDIA GeForce GTX 2080 Ti.

**Table A1:** Hyperparameters for IsotopeNet and U-Net.

Parameter	IsotopeNet	U-Net
Optimizer	Adam [29]	Adam [29]
Learning rate	0.005	0.0005
LR scheduler	0.8 every 10 epochs	0.8 every 5 epochs
Weight decay	0.01	0
Dropout	0.5	0
Batch size	32	64
Max. number of epochs	30	50

## Appendix B. Results

**Table A2:** Evaluation of classification with different MALDI networks on the **spectral level**, on the respective training and validation dataset. Balanced accuracy (Bal. accuracy) is the arithmetic mean of the sensitivities of both classes. Note that in a two-class classification problem, the sensitivity of one class corresponds to the specificity of the other. The mean and standard deviation of five identical training processes are displayed in brackets. The network with the highest validation accuracy was chosen.

	Bal. Accuracy	Sensitivity (SqCC)	Sensitivity (ADC)
	**MALDI1**		
**Training**	0.982 (0.983 ± 0.004)	0.984 (0.979 ± 0.008)	0.981 (0.987 ± 0.005)
**Validation**	0.986 (0.983 ± 0.002)	0.992 (0.995 ± 0.005)	0.981 (0.972 ± 0.008)
	**MALDI2**		
**Training**	0.986 (0.958 ± 0.038)	0.981 (0.938 ± 0.045)	0.991 (0.977 ± 0.035)
**Validation**	0.978 (0.953 ± 0.015)	0.969 (0.953 ± 0.029)	0.986 (0.954 ± 0.032)
	**MALDI3**		
**Training**	0.992 (0.981 ± 0.013)	0.988 (0.973 ± 0.027)	0.995 (0.989 ± 0.008)
**Validation**	0.979 (0.967 ± 0.012)	0.975 (0.967 ± 0.011)	0.983 (0.967 ± 0.020)

**Table A3:** Confusion matrices of classification with different MALDI networks on the **core/whole section level,** on the respective **training** and **validation dataset** (Pred. = Prediction). Section/core-wise classification based on the majority vote (***p* = 0.5**). Diagonal elements of the matrices show the number of correctly classified cores/sections, off-diagonal elements show number of misclassified cores/whole sections.

		Training (Cores)		Validation (Whole Sections)	
	True/Pred.	SqCC	ADC	SqCC	ADC
**MALDI1**	SqCC	217	6	7	0
	ADC	4	175	0	7
**MALDI2**	SqCC	219	4	7	0
	ADC	3	176	0	7
**MALDI3**	SqCC	214	7	7	0
	ADC	3	175	0	7

**Table A4:** Confusion matrices of classification with different MALDI networks on different **test datasets** on the **section level** (Pred. = Prediction). Section-wise classification based on the majority vote (***p* = 0.5**). The diagonal elements of the matrices show the number of correctly classified sections, off-diagonal elements show the number of misclassified sections. Explanation of different datasets can be found in Table 2.

		MALDI1		MALDI2		MALDI3	
	True/Pred.	SqCC	ADC	SqCC	ADC	SqCC	ADC
Test Dataset 1	SqCC	8	0	8	0	8	0
	ADC	0	8	0	8	0	8
Test Dataset 2	SqCC	7	1	7	1	7	1
	ADC	0	8	0	8	0	8
Test Dataset 3	SqCC	7	1	7	1	7	1
	ADC	0	8	0	8	0	8
Test Dataset 4	SqCC	7	1	6	2	7	1
	ADC	0	8	0	8	1	7
